# A quantitative analysis linking seabird mortality and marine debris ingestion

**DOI:** 10.1038/s41598-018-36585-9

**Published:** 2019-03-01

**Authors:** Lauren Roman, Britta Denise Hardesty, Mark A. Hindell, Chris Wilcox

**Affiliations:** 1 CSIRO Oceans and Atmosphere, Hobart, Tasmania Australia; 2 Institute for Marine and Antarctic Studies, University of Tasmania, Hobart, Tasmania Australia; 3 Antarctic Climate and Ecosystems CRC, University of Tasmania, Hobart, Tasmania Australia

## Abstract

Procellariiformes are the most threatened bird group globally, and the group with the highest frequency of marine debris ingestion. Marine debris ingestion is a globally recognized threat to marine biodiversity, yet the relationship between how much debris a bird ingests and mortality remains poorly understood. Using cause of death data from 1733 seabirds of 51 species, we demonstrate a significant relationship between ingested debris and a debris-ingestion cause of death (dose-response). There is a 20.4% chance of lifetime mortality from ingesting a single debris item, rising to 100% after consuming 93 items. Obstruction of the gastro-intestinal tract is the leading cause of death. Overall, balloons are the highest-risk debris item; 32 times more likely to result in death than ingesting hard plastic. These findings have significant implications for quantifying seabird mortality due to debris ingestion, and provide identifiable policy targets aimed to reduce mortality for threatened species worldwide.

## Introduction

Pollution of the world’s oceans by anthropogenic marine debris is a global problem^1^. With 250 000 tonnes of marine debris afloat currently, our mismanaged waste presents a ubiquitous threat to marine wildlife^1^. Ingestion of buoyant marine debris in the ocean is a widespread, emerging threat to seabirds^2,3^, particularly so for albatrosses and petrels (Procellariiformes)^4^, which can mistake the floating trash for food^4,5^. Seabirds are the world’s most threatened group of birds, with nearly half of species experiencing population declines, and 28% threatened globally^6^. Presently, half of the world’s seabird species ingest marine debris^7^, with the greatest expected adverse effects occurring in Australasia, at the Southern Ocean boundary of the Tasman sea^8^ where the highest global seabird biodiversity occurs^9^.

Significant declines in Australasia’s albatross and petrel populations are driven by a number of threats^10^, but the contribution of marine debris ingestion is unknown. At least 51 of Australasia’s Procellariiform species ingest marine debris^11^, and likely more species ingest marine debris, though it has not been documented^10^. Ninety nine percent of all seabird species are predicted to ingest marine debris by 2050^8^. The ubiquity of debris ingestion among threatened and declining seabirds highlights the need to quantify the level of threat that it poses to seabirds. Quantifying the effects of the ingestion of marine debris on individual mortality, and ultimately on wild populations, is one of the primary research priorities in marine debris research^12^.

While there are observations of seabird mortalities resulting from the ingesting debris^13^, and anecdotal evidence that ingestion has sublethal and lethal impacts on seabirds, a quantitative relationship is yet to be established. This is due to the difficulty of establishing a dose-response relationship between ingestion and mortality. In the absence of experimental feeding trials, the necropsy of wild seabirds collected deceased can provide the data to estimate a dose-response relationship. Wild seabirds die for many reasons including starvation, disease, injury, fisheries by-catch, and the ingestion of marine debris. We used seabirds that had an identifiable cause of death (e.g. fisheries, by-catch or advanced disease) as a control group (assuming their death was random with respect to the ingestion of marine debris). We compared these birds that died of indeterminate causes to those that were identified as having died due to marine debris ingestion (gut blockage, perforation or impaction). With this information, we investigated whether the ingested debris load is lowest in seabirds dying due to non-marine debris related causes, increasing for seabirds with indeterminate causes of death (which could potentially have died due to debris ingestion), and highest in seabirds that died from ingesting marine debris. Ultimately, we also used all seabirds in the dataset to estimate the relationship between the probability of death due to marine ingestion and the load of ingested marine debris. With this information we aim to determine whether there is a dose-response relationship between marine debris ingestion and seabird lifetime mortality, and if so, to predict the relationship between the load of ingested marine debris and the probability of death due to marine debris ingestion.

## Results

Of the 1733 seabirds examined, 557 (32.1%) had ingested marine debris, ranging from 1–40 items, with a maximum weight of 3440 mg and volume of 3621 mm^3^. Weight and volume of ingested debris was not recorded for 27 of the debris samples. The type of debris ingested was not recorded for 17 of the debris samples. In total, 2671 items of known type were collected. Hard plastic, both fragments and pellets, were most common, accounting for 92.4% of all items ingested. The remaining items ingested included soft plastics such as packaging (2.1%), balloon fragments (2%), rubbers and foams including polystyrene, expanded polyethylene and other synthetic foams (1.3%), rope and rope fibers (1%), fishing related rubbish (0.7%) with other debris types contributing 0.5%.

**Figure 1:**
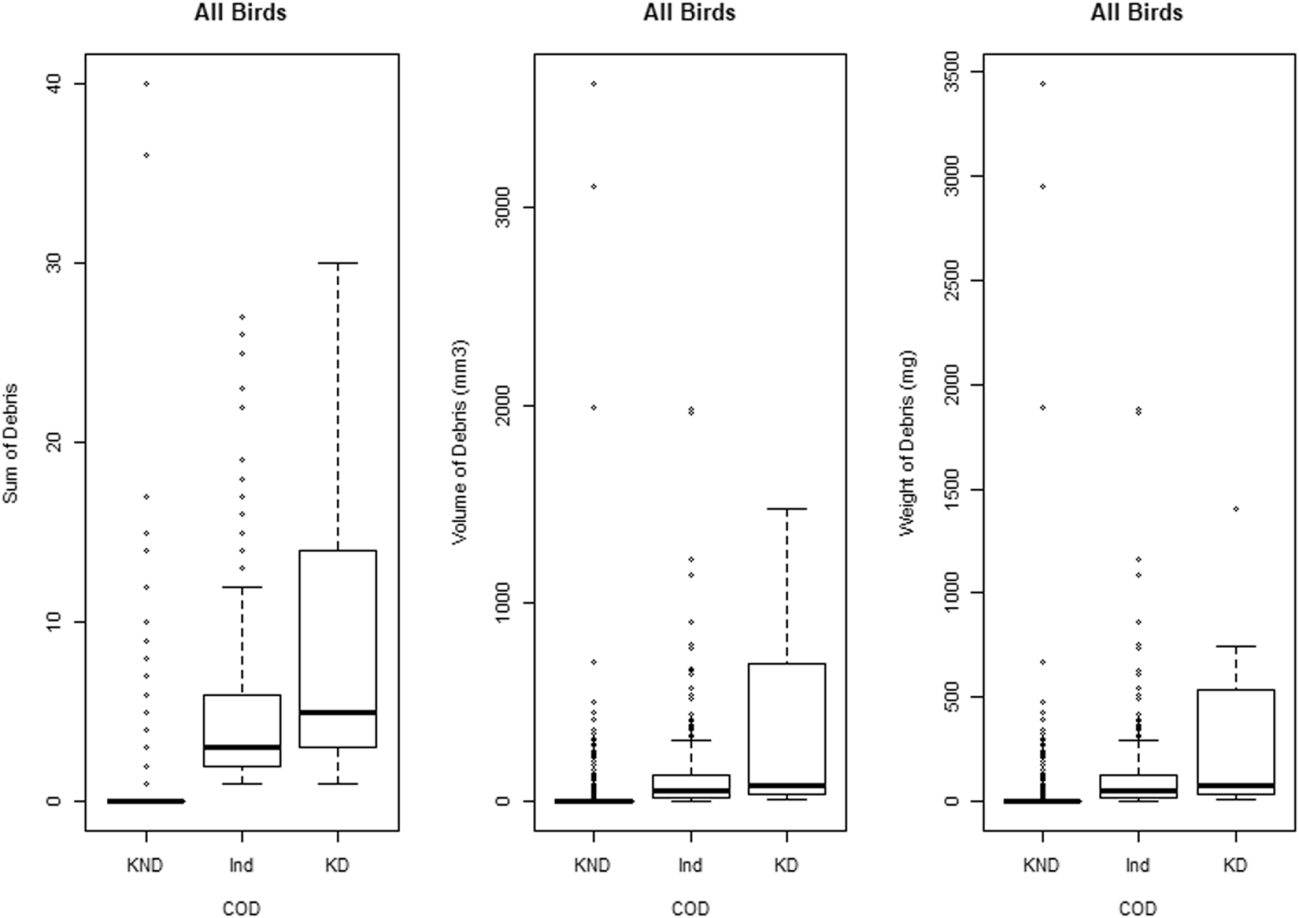
Quantity of marine debris ingested by seabirds by cause of death. The sum (left), cumulative weight (middle), and volume (right) of marine debris items ingested by Procellariiformes that have died from non-debris (KND) related causes, indeterminate (Ind) causes, and as a result of their marine debris ingestion (KD). An analysis of variance (ANOVA) and post-hoc pairwise t-test demonstrate that the sum of debris, volume of debris and weight of debris differ significantly between KND, Ind and KD birds.

**Figure 2:**
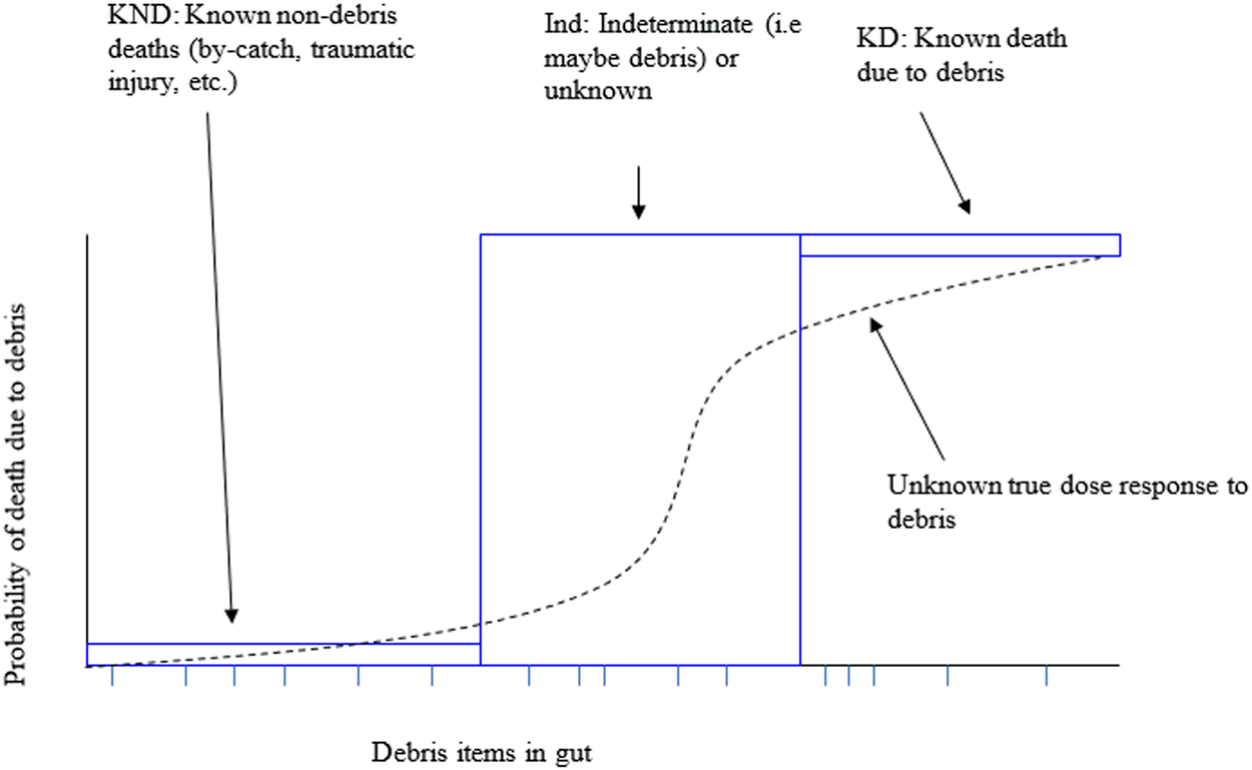
Theoretical dose-response relationship between marine debris and seabird death. This model is adapted from Wilcox *et al*.^14^. We assigned each seabird death into three cause of death categories: (1) known, non-debris ingestion related (KND), where there was a clearly identifiable cause such as drowning as fisheries by-catch; (2) indeterminate (Ind), where there was marine debris present in the gut but also other possible causes, such as starvation and (3) known, marine debris ingestion related (KD), where there was a gut blockage, or other strong evidence of the ingested debris being the cause of mortality.

The cause of death of 1265 (73%) of the seabirds was not debris ingestion (KND). Thirteen birds died as a result of marine debris ingestion (KD); five fairy prions, *Pachyptila turtur*, four short-tailed shearwaters, *Ardenna tenuirostris*, one Salvin’s prion, *Pachyptila salvini*, one Antarctic prion, *Pachyptila desolata*, one blue petrel, *Halobaena caerulea*, and one light-mantled sooty albatross, *Phoebetria palpebrata*. Blockage of the gastrointestinal tract was the leading cause of mortality (7 birds), followed by obstruction of the gastrointestinal tract (5 birds) causing infection or other complications, and one perforated gut. The site of blockage and obstruction was the isthmus between the proventriculus and the gizzard in 8 birds, the gizzard in two birds and the entrance to the small intestine in two birds. The perforation of the gut occurred in the proventriculus. The items causing death were hard plastics (n = 7), balloons (n = 3) and expanded foam (n = 2). The gut perforation was caused by plastic strapping. A further 9 birds; four short-tailed shearwaters, two slender-billed prions, *Pachyptila belcheri*, one Salvin’s prion, one white-faced storm-petrel, *Pelagodroma marina*, and one southern fulmar, *Fulmarus glacialoides*, were deemed very likely to have died from marine debris due to obstruction or blockage of the GI tract, but this could not be confirmed due to the decomposition of the bird. The obstructing items were hard plastic (n = 4), balloons (n = 2), soft plastic packaging (n = 2) and synthetic rope (n = 1). These birds were allocated an indeterminate cause of death (Ind) for the purpose of this model. The remaining 446 birds were allocated an indeterminate cause of death (Ind). The number of items ingested by seabirds ranked according to the cause of death was: Known (non-marine debris related) <Indeterminate (possibly marine debris related) <known (marine debris related) (Fig. 1), following the theoretical model (Fig. 2). Seabirds that died from marine debris had significantly higher numbers of ingested marine debris than birds with indeterminate causes of death and those known to have died from other causes (P < 0.05).

The best model, based on AIC, for the relationship between the number of debris items in the gut and the cause of death includes a main effect for species and the species weight (Table 1), having an AIC of 3757.7.

**Table 1:** AIC table of explanatory factors driving quantity of ingested debris.

Models	AICs	AICs model
1	COD + species + species weight	3757.7
2	COD + species	3759.7
3	COD + age + species	3768.1
4	COD + age + family + species weight	4140.8
5	COC + age + family	4142.1
6	COD + family + species weight	4142.3
7	COD + family	4145.7
8	COD + species weight	4235.1
9	COD + age	4275.4
10	COD	4277.4
11	Null	4918.2

AIC table of models run to explain the sum of debris items ingested with cause of death (COD) and individual factors such as species, age, family and average species weight. Note that all models are an improvement over the null model (model 11), with COD, species and species weight as the best model.

**Figure 3:**
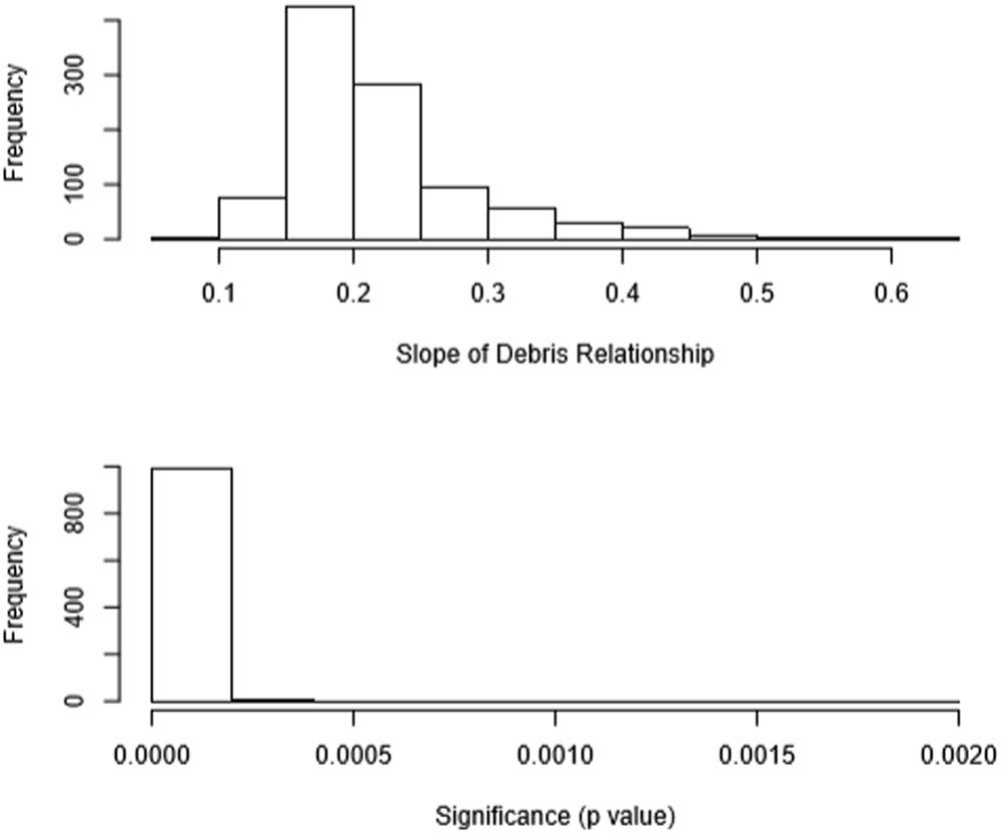
Slope of the relationship between probability of death due to marine debris ingestion and the debris load in the seabird. The top plot shows the distribution of slope estimates for the number of debris items in the gut, the lower plot shows the significance of these coefficients, from 1,000 Monte Carlo regression analysis samples.

**Figure 4:**
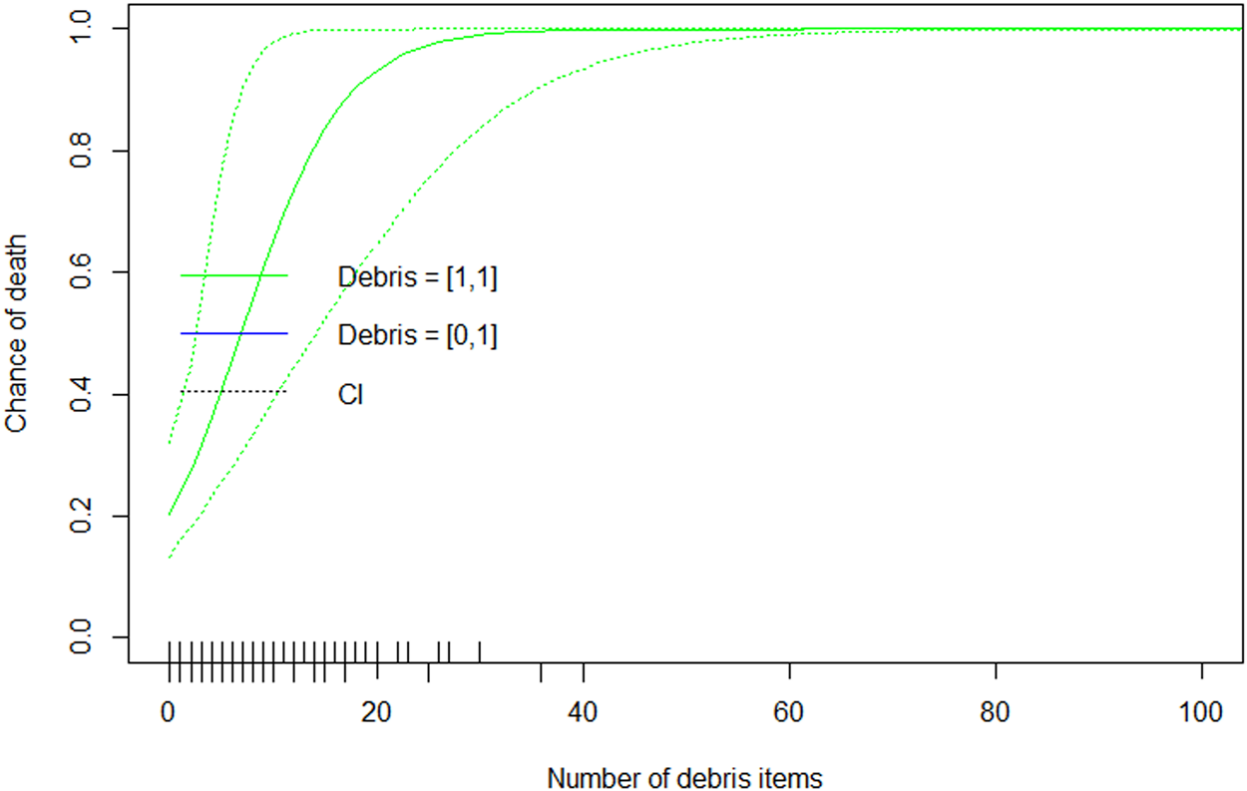
Probability of mortality due to marine debris ingestion with increasing ingested marine debris load. Model results are based on the seabird species weight. Two models are shown, one based on Monte Carlo simulations. The first model assumes the cause of death has been assigned correctly, leading to animals with plastic ingestion as an assigned cause having a probability in the interval [1, 1] in the Monte Carlo process. The second model assumes plastic has been assigned incorrectly, leading to a probability in the interval [0, 1]. For each model we show the median (solid) and the extreme values (dotted) over 1,000 Monte Carlo simulations. The rug plot along the bottom of the figure shows the number of marine debris items ingested by each of the seabirds in our samples, with top showing seabirds that died of known non-debris ingestion causes, and beneath indicating those that died of either debris ingestion or were indeterminate.

The relationship between the number of ingested items and the probability of death due to debris ingestion had a significantly positive slope term (Fig. 3). Using the median values of the regression parameters from the Monte Carlo analysis, and the average species weight of the species, we were able to predict the relationship between the load of ingested marine debris and the probability of death due to marine debris ingestion (Fig. 4). Species was not included in this model as our data did not include KND, Ind and KD individuals across all species. Our model shows that a bird with one ingested debris item has a 20.4% chance of mortality, rising through 50% chance of mortality at 9 items and 100% at 93 items. Using the bounds of this relationship across all estimated values from our Monte Carlo simulations, we found a relatively small amount of uncertainty (Fig. 4).

## Discussion

The marine debris load was lowest in seabirds dying due to non-marine debris related causes, rising through indeterminate causes of death and was highest in seabirds that died from marine debris ingestion. Seabirds that died of debris ingestion had, on average, greater marine debris loads in their gastro-intestinal tracts. This supports similar findings in sea turtles that the causes of death are segregated in terms of plastic concentration^14^. Secondly, when we modelled the probability of death due to marine debris ingestion, we found a positive relationship, confirming that larger loads of marine debris items in the gastro-intestinal tract lead to a higher probability of mortality. In this study each seabirds’ mortality and ingested debris load observed over an unknown period of its lifetime. We do not know either the birds age or turnover rate for the ingested debris loads, and assume the debris load we recorded at death represents an average load and turnover that lead to the death for the bird. These results can be used to predict the lifetime mortality rate for seabirds where the load of marine debris items is known. It is interesting to note that a bird has a 20.4% chance of mortality with a single ingested marine debris item, a statistic supported by two individuals in our study having died as a result of having ingested a single item causing obstruction, in one case a knotted balloon obstructing the entrance to the intestine, and the second, a hard plastic blockage in the isthmus juncture.

Some Procellariiform species, including short-tailed shearwater, slender-billed prion, Salvin’s prion and white-faced storm petrels have a median debris ingestion of 1 or greater^11^, and this model provides a valuable quantitative framework to modelling population impacts on Procellariiform seabirds, especially species with high incidences of marine debris ingestion^11^.

All but one death resulted from obstruction in one of three locations; the gizzard and the small junctures leading in and out of the gizzard, causing either a large obstruction through to a complete blockage. The unique gizzard morphology of Procellariiform seabirds, (the gizzard is separated from the proventriculus by an isthmus juncture where hard items can become lodged and not easily regurgitated) puts Procellariiformes at higher risks of obstructions^15^. For this reason we can expect Procellariiformes to be at higher risk of mortality by marine debris than other species.

The composition of the marine debris influenced the probability of the likelihood of mortality, as observed in sea turtles. In turtles small hard plastic fragments may pass quickly through the gut with little incident^14^, but soft plastics are more likely to compact and contribute to a blockage or obstruction^16^. In seabirds not all ingested debris posed an equal risk of mortality. Hard plastics were responsible for only half (55%) of known and probable seabird deaths, but were the vast majority of items ingested. Soft plastic packaging, balloon fragments, rubbers and synthetic foams together accounted for only 5.4% of items but were responsible for 42% of probable and known mortalities. The ingestion of a soft item (10 confirmed or probable deaths from 140 items ingested) is 14 times more likely to result in death than the ingestion of a hard item (12 known/probable deaths from 2468 items ingested). This may be due to soft and pliable items resisting peristalsis and becoming obstructions^17^. Obstructions of soft pliable synthetics, including plastics and rubbers, have been recorded in a number of species including dogs^18^, cattle^17^ and birds^19^. In birds, such obstructions can cause death by enteritis, as well blocking the passage of food resulting in starvation^19^.

Balloons were the marine debris most likely to cause mortality. Where ingestion of balloons or balloon fragments were found, these fragments were the known or probable cause of death in 18.5% of balloon ingesting seabirds, with the ingestion of a balloon or balloon fragment is 32 times more likely to result in death than ingestion of a hard plastic fragment (5 known/probable deaths from 32 balloons ingested). Other studies have highlighted balloons as a high risk items for ingestion in other taxa^20,21^. Of particular concern is that seabirds may select for balloons when foraging because of their resemblance to prey, especially squid^4^. All balloons in this study were ingested by species that eat squid, suggesting these squid-feeding species are likely to have higher mortality rates. We suggest that reducing the presence of balloons and balloon fragments in the ocean would directly reduce seabird mortalities resulting from marine debris ingestion, and would have eliminated the 23% of confirmed KD deaths in this study for which balloons were cause. We propose that the most immediate solution to reduce seabird mortality from anthropogenic marine debris ingestion would be to reduce the amount of marine debris, particularly the number of balloons, entering the ocean.

In summary, we provide strong evidence that marine debris ingestion can cause death in seabirds, and that the probability of death increases with the number of items ingested. This finding has substantial transboundary implications for estimating mortality due to marine debris ingestion and consequently managing wildlife population declines. In addition, we highlight high-risk marine debris items, providing identifiable policy targets aimed at reducing mortality caused by high-risk debris items. Reducing the input of waste into the environment, in particular high-risk items, will undoubtedly reduce debris ingestion mortality in marine wildlife.

## Methods

### Material collection

Data for this study included deceased Procellariiform seabirds obtained as fisheries by-catch, veterinary casualties and beach-washed carcasses collected from Australia and New Zealand. In Australia, Fraser Island, QLD, was the most northerly collection point, and Macquarie Island the most southerly. Collection spanned the width of the country from Ballina, NSW in the east and Perth, WA in the West. Collection in New Zealand spanned the country North (Bay of Plenty) to South (Invercargill area) and included by-catch oceanic regions between the south of the continent and the Auckland Islands to the south.

In total, 1733 individuals of 51 species were collected and necropsied following Van Franekers collection and dissection procedures^22^, and cause of death was determined. Birds with a cause of death unrelated to the ingestion of marine debris (fisheries by-catch, some veterinary casualties, injury, disease/infection) were assigned the category “non-debris” cause of death. Seabirds with ingestion of marine debris resulting in clear gut impaction, perforation or blockage, (often with associated local signs of infection and irritation and/or undigested food blocked from passing through the gut), were assigned to category “debris” cause of death. In these cases, the object responsible for the death was determined by its association with the site of lesion. Seabirds where debris was present but the cause of death could not be positively determined were assigned an ‘indeterminant’ cause of death. When gut blockage or impaction was suspected but could not be positively confirmed as the cause of death, the cause of death was also ruled as indeterminate. As a result, the birds deemed as having died from ingested marine debris is conservative and likely lower than the actual number.

Marine debris removed from the seabirds were rinsed, dried, weighed and the rigid objects (mostly fragments of hard marine debris) were measured. The length, width and depth of each item was measured at the longest edge. Volumes were calculated by multiplying the weight by an average density value for the material type; 0.95 g per cm^3^ for hard plastics^23,24^, by 0.91 g per cm^3^ for balloons^25^ and 7.7 g per cm^3^ for fishing hooks^26^. The volume of other items, including large quantities of soft plastic, rubbers and expanded foams, were measured per 1 ml of water displacement.

#### Data preparation

To examine the dose-response relationship between marine debris and seabird death, we assigned each seabird death into three cause of death categories, as described above: (1) known, non-debris ingestion related (*KND*), where there was a clearly identifiable cause such as drowning as fisheries by-catch; (2) indeterminate (*Ind*), where there was marine debris present in the gut but also other possible causes, such as starvation and (3) known, marine debris ingestion related (*KD*), where there was a gut blockage, or other strong evidence of the ingested debris being the cause of mortality. If debris ingestion results in death, we expect that the number of ingested debris items ingested should scale as: KND < Ind < KD. This slope of the proposed relationship is shown in Fig. 2, and is adapted from Wilcox *et al*.^14^.

#### Statistical analysis

Statistical analyses were performed using R (Version 3.3.3)^27^, following Wilcox *et al*.^14^. We tested for variation in the count of ingested marine debris items present in individual birds within the three cause of death categories using a generalized linear regression model (GLM), using a negative binomial error, due to over-dispersion in the data, which proved adequate based on a Chi square test. In addition to cause of death, we included species, age, average species weight and taxonomic family variables, as these can influence the frequency of debris ingestion^11^. We selected the best model using the Akaike Information Criterion (AIC) (Table 1), which was used to estimate the pairwise differences between the coefficients for cause of death to determine whether they followed the expected KND < Ind < KD order (Fig. 2).

An interval value was assigned for the probability of death due to marine debris ingestion for each bird. Birds with known causes of death other than debris ingestion (KND) were assigned [0, 0], birds with deaths caused by debris ingestion (KD) were assigned [1, 1]. Birds with indeterminate causes of death were assigned the range [0, 1]. We performed a logistic regression to relate the probability of death due to debris ingestion to the number of debris items in an animal’s gut. A Monte Carlo technique was used to accommodate the interval values for indeterminate causes of death, randomly drawing a value in the interval [0, 1] for each bird in the indeterminate cause of death category to fit the model to the full dataset across all three causes of death. We captured the estimated coefficient for the number of marine debris items in the gut and its standard error. This process was repeated 1,000 times. The output coefficient and its significance for each fit was used for each iteration to create an expected distribution. We accounted for the effect of gut volume on the relationship between chance of death due to marine debris and the number of items by including the average species weight of the bird, as per Marchant and Higgins^28^ as a covariate, on the assumption that species weight is proportional to gut volume.

## Electronic supplementary material

Supplementary information
