# Supplementary material for: A quantitative analysis linking seabird mortality and marine debris ingestion
Source: Sci Rep. 2019 Mar 1;9:3202. doi: 10.1038/s41598-018-36585-9 (PMC6397299; doi:10.1038/s41598-018-36585-9)
Supplement: Supplementary file 1 — Supplementary information [file 41598_2018_36585_MOESM1_ESM.docx]

**Supplementary information**

**A quantitative analysis linking seabird mortality and marine debris ingestion**

Lauren Roman^1*^, Britta Denise Hardesty^2^, Mark A. Hindell^1,3^, Chris Wilcox^2^

1 Institute for Marine and Antarctic Studies, University of Tasmania, Hobart, Tasmania, Australia,

2 CSIRO Oceans and Atmosphere, Hobart, Tasmania, Australia,

3. Antarctic Climate and Ecosystems CRC, University of Tasmania, Hobart, Tasmania, Australia,

* lauren.roman@utas.edu.au

**Supplementary Information** **Table 1**: The incidence of marine debris ingestion in Australasian seabird species, and the frequency of items examined. ‘N’ is the number of individuals that were examined for debris ingestion, ‘N/w’ is the number of individual birds with ingestion of the specific item, and ‘tot’ is the total sum of the items ingested.

| Species | N | N/w debris | N/w hard plas | Tot hard plas | N/w balloon | Tot balloon | N/w soft plas | Tot soft plas | N/w foam | Tot foam | N/w rope | Tot rope | N/w fishing | Tot fishing | N/w other | Tot other |
| --- | --- | --- | --- | --- | --- | --- | --- | --- | --- | --- | --- | --- | --- | --- | --- | --- |
| Albatrosses |  |  |  |  |  |  |  |  |  |  |  |  |  |  |  |  |
| Antipodean albatross | 1 | 0 | 0 | 0 | 0 | 0 | 0 | 0 | 0 | 0 | 0 | 0 | 0 | 0 | 0 | 0 |
| Black-browed albatross | 9 | 1 | 1 | 1 | 0 | 0 | 0 | 0 | 0 | 0 | 0 | 0 | 0 | 0 | 0 | 0 |
| Buller's albatross | 90 | 1 | 0 | 0 | 0 | 0 | 0 | 0 | 0 | 0 | 0 | 0 | 0 | 0 | 1 | 2 |
| Campbell albatross | 4 | 0 | 0 | 0 | 0 | 0 | 0 | 0 | 0 | 0 | 0 | 0 | 0 | 0 | 0 | 0 |
| Chatham Island albatross | 1 | 0 | 0 | 0 | 0 | 0 | 0 | 0 | 0 | 0 | 0 | 0 | 0 | 0 | 0 | 0 |
| Gibson's albatross | 2 | 0 | 0 | 0 | 0 | 0 | 0 | 0 | 0 | 0 | 0 | 0 | 0 | 0 | 0 | 0 |
| Grey-headed albatross | 4 | 1 | 1 | 1 | 1 | 2 | 0 | 0 | 0 | 0 | 0 | 0 | 0 | 0 | 1 | 1 |
| Light-mantled sooty albatross | 6 | 1 | 0 | 0 | 1 | 1 | 0 | 0 | 0 | 0 | 0 | 0 | 0 | 0 | 0 | 0 |
| New Zealand white-capped albatross | 85 | 0 | 0 | 0 | 0 | 0 | 0 | 0 | 0 | 0 | 0 | 0 | 0 | 0 | 0 | 0 |
| Salvin's albatross | 30 | 0 | 0 | 0 | 0 | 0 | 0 | 0 | 0 | 0 | 0 | 0 | 0 | 0 | 0 | 0 |
| Shy albatross | 25 | 1 | 0 | 0 | 0 | 0 | 1 | 1 | 0 | 0 | 0 | 0 | 0 | 0 | 0 | 0 |
| Shy Albatross | 1 | 0 | 0 | 0 | 0 | 0 | 0 | 0 | 0 | 0 | 0 | 0 | 0 | 0 | 0 | 0 |
| Sooty albatross | 1 | 0 | 0 | 0 | 0 | 0 | 0 | 0 | 0 | 0 | 0 | 0 | 0 | 0 | 0 | 0 |
| Southern royal albatross | 2 | 0 | 0 | 0 | 0 | 0 | 0 | 0 | 0 | 0 | 0 | 0 | 0 | 0 | 0 | 0 |
| Wandering albatross | 5 | 0 | 0 | 0 | 0 | 0 | 0 | 0 | 0 | 0 | 0 | 0 | 0 | 0 | 0 | 0 |
| Diving petrels | | | | | | | | | | | | | | | | |
| Common diving petrel | 31 | 2 | 2 | 3 | 0 | 0 | 0 | 0 | 0 | 0 | 0 | 0 | 0 | 0 | 0 | 0 |
| South Georgian diving petrel | 1 | 0 | 0 | 0 | 0 | 0 | 0 | 0 | 0 | 0 | 0 | 0 | 0 | 0 | 0 | 0 |
| Fulmarine petrels | | | | | | | | | | | | | | | | |
| Antarctic petrel | 1 | 1 | 1 | 2 | 0 | 0 | 0 | 0 | 0 | 0 | 0 | 0 | 0 | 0 | 0 | 0 |
| Cape petrel | 7 | 3 | 3 | 18 | 0 | 0 | 1 | 2 | 0 | 0 | 0 | 0 | 0 | 0 | 0 | 0 |
| Southern fulmar | 4 | 3 | 2 | 10 | 1 | 2 | 1 | 1 | 0 | 0 | 0 | 0 | 0 | 0 | 0 | 0 |
| Gadfly petrels | | | | | | | | | | | | | | | | |
| Black-winged petrel | 2 | 0 | 0 | 0 | 0 | 0 | 0 | 0 | 0 | 0 | 0 | 0 | 0 | 0 | 0 | 0 |
| Cook's petrel | 7 | 1 | 1 | 1 | 0 | 0 | 0 | 0 | 0 | 0 | 1 | 1 | 0 | 0 | 0 | 0 |
| Gould's petrel | 5 | 3 | 3 | 5 | 0 | 0 | 0 | 0 | 0 | 0 | 0 | 0 | 0 | 0 | 0 | 0 |
| Great-winged petrel | 4 | 0 | 0 | 0 | 0 | 0 | 0 | 0 | 0 | 0 | 0 | 0 | 0 | 0 | 0 | 0 |
| Grey-faced petrel | 4 | 0 | 0 | 0 | 0 | 0 | 0 | 0 | 0 | 0 | 0 | 0 | 0 | 0 | 0 | 0 |
| Kerguelen petrel | 3 | 1 | 1 | 1 | 0 | 0 | 0 | 0 | 0 | 0 | 0 | 0 | 0 | 0 | 0 | 0 |
| Mottled petrel | 3 | 1 | 1 | 1 | 0 | 0 | 0 | 0 | 0 | 0 | 0 | 0 | 0 | 0 | 0 | 0 |
| Providence petrel | 1 | 0 | 0 | 0 | 0 | 0 | 0 | 0 | 0 | 0 | 0 | 0 | 0 | 0 | 0 | 0 |
| White-headed petrel | 6 | 1 | 0 | 0 | 0 | 0 | 1 | 1 | 0 | 0 | 0 | 0 | 0 | 0 | 0 | 0 |
| White-necked petrel | 1 | 0 | 0 | 0 | 0 | 0 | 0 | 0 | 0 | 0 | 0 | 0 | 0 | 0 | 0 | 0 |
| Giant petrels | | | | | | | | | | | | | | | | |
| Northern giant petrel | 4 | 2 | 2 | 6 | 0 | 0 | 0 | 0 | 0 | 0 | 0 | 0 | 0 | 0 | 0 | 0 |
| Southern giant petrel | 8 | 5 | 5 | 41 | 2 | 2 | 0 | 0 | 0 | 0 | 0 | 0 | 0 | 0 | 0 | 0 |
| Prions | | | | | | | | | | | | | | | | |
| Antarctic prion | 17 | 12 | 12 | 63 | 0 | 0 | 2 | 2 | 2 | 3 | 0 | 0 | 0 | 0 | 0 | 0 |
| Blue petrel | 1 | 1 | 1 | 29 | 0 | 0 | 0 | 0 | 0 | 0 | 0 | 0 | 0 | 0 | 0 | 0 |
| Blue Petrel | 1 | 0 | 0 | 0 | 0 | 0 | 0 | 0 | 0 | 0 | 0 | 0 | 0 | 0 | 0 | 0 |
| Broad-billed prion | 14 | 3 | 3 | 10 | 0 | 0 | 0 | 0 | 0 | 0 | 0 | 0 | 0 | 0 | 0 | 0 |
| Fairy prion | 236 | 61 | 55 | 131 | 2 | 2 | 1 | 4 | 2 | 6 | 1 | 1 | 3 | 3 | 2 | 3 |
| Salvin's prion | 24 | 17 | 16 | 63 | 1 | 1 | 1 | 1 | 0 | 0 | 1 | 2 | 0 | 0 | 0 | 0 |
| Slender-billed prion | 79 | 49 | 49 | 187 | 3 | 5* (4) | 5 | 6 | 0 | 0 | 5 | 7 | 0 | 0 | 0 | 0 |
| Procellarine petrels | | | | | | | | | | | | | | | | |
| Black petrel | 7 | 0 | 0 | 0 | 0 | 0 | 0 | 0 | 0 | 0 | 0 | 0 | 0 | 0 | 0 | 0 |
| Grey petrel | 7 | 0 | 0 | 0 | 0 | 0 | 0 | 0 | 0 | 0 | 0 | 0 | 0 | 0 | 0 | 0 |
| Tahiti petrel | 1 | 0 | 0 | 0 | 0 | 0 | 0 | 0 | 0 | 0 | 0 | 0 | 0 | 0 | 0 | 0 |
| Westland petrel | 16 | 2 | 1 | 1 | 1 | 1 | 0 | 0 | 1 | 1 | 0 | 0 | 0 | 0 | 0 | 0 |
| White-chinned petrel | 221 | 2 | 1 | 1 | 0 | 0 | 0 | 0 | 0 | 0 | 0 | 0 | 0 | 0 | 0 | 0 |
| Shearwaters | | | | | | | | | | | | | | | | |
| Flesh-footed shearwater | 213 | 55 | 50 | 198 | 2 | 2 | 0 | 0 | 0 | 0 | 0 | 0 | 4 | 7 | 2 | 4 |
| Fluttering shearwater | 70 | 7 | 4 | 5 | 1 | 1 | 0 | 0 | 0 | 0 | 3 | 5 | 0 | 0 | 0 | 0 |
| Hutton's shearwater | 4 | 0 | 0 | 0 | 0 | 0 | 0 | 0 | 0 | 0 | 0 | 0 | 0 | 0 | 0 | 0 |
| Little shearwater | 8 | 4 | 4 | 7 | 0 | 0 | 0 | 0 | 0 | 0 | 1 | 1 | 0 | 0 | 1 | 1 |
| Short-tailed shearwater | 332 | 287 | 273 | 1456 | 12 | 34* (14) | 16 | 38 | 15 | 21 | 7 | 11 | 5 | 8 | 5 | 5 |
| Sooty shearwater | 89 | 19 | 17 | 137 | 0 | 0 | 0 | 0 | 0 | 0 | 0 | 0 | 0 | 0 | 0 | 0 |
| Wedge-tailed shearwater | 28 | 4 | 3 | 12 | 0 | 0 | 0 | 0 | 0 | 0 | 0 | 0 | 1 | 1 | 0 | 0 |
| Storm petrels | | | | | | | | | | | | | | | | |
| Black-bellied storm petrel | 1 | 0 | 0 | 0 | 0 | 0 | 0 | 0 | 0 | 0 | 0 | 0 | 0 | 0 | 0 | 0 |
| White-faced storm petrel | 6 | 6 | 6 | 78 | 0 | 0 | 0 | 0 | 0 | 0 | 0 | 0 | 0 | 0 | 0 | 0 |
| Grand total | 1733 | 557 | 518 | 2468 | 27 | 53* (32) | 29 | 56 | 20 | 31 | 19 | 28 | 13 | 19 | 12 | 16 |

*This number reflects the number of large fragments counted. Where a number of fragments appeared to result from fragmentation of a single balloon, predicted number of original balloons ingested appear in brackets.

**Supplementary Information Table 2:** Occurrences where marine debris ingestion was a known or likely cause of death. Thirteen birds were determined to have died as a result of marine debris ingestion (KD). A further nine birds suspected to have died due to debris ingestion, but deaths couldn’t be allocated with confidence, were marked as indeterminate (Ind).

| Bird Group | Species | Item causing death | Item location | Cause of death |
| --- | --- | --- | --- | --- |
| Known death due to marine debris ingestion (KD) | | | | |
| Prions | Fairy prion | Hard plastic | Entry to intestines | Complete blockage |
| Prions | Fairy prion | Rubber/expanded foam | Gizzard | Obstruction of gizzard |
| Prions | Fairy prion | Hard plastic | Isthmus | Complete blockage |
| Prions | Fairy prion | Hard plastic | Isthmus | Complete blockage |
| Prions | Fairy prion | Hard plastic | Isthmus | Complete blockage |
| Prions | Antarctic prion | Hard plastic | Isthmus | Obstruction |
| Prions | Salvin's prion | Hard plastic | Opening between gizzard and intestine | Obstruction |
| Prions | Blue petrel | Hard plastic | Isthmus and gizzard | Obstruction |
| Shearwaters | Short-tailed shearwater | Rubber/expanded foam | Proventriculus and isthmus | Complete blockage |
| Shearwaters | Short-tailed shearwater | Balloon | Proventriculus and isthmus | Complete blockage |
| Shearwaters | Short-tailed shearwater | Hard plastic | Proventriculus | Perforation |
| Shearwaters | Short-tailed shearwater | Balloon | Proventriculus and isthmus | Obstruction |
| Albatrosses | Light-mantled sooty albatross | Balloon | Opening between gizzard and intestine | Obstruction |
| Suspected death due to marine debris ingestion, but recorded as indeterminate (Ind) | | | | |
| Prions | Slender-billed prion | Hard plastic | Gizzard | Obstruction of gizzard |
| Prions | Slender-billed prion | Balloon | Gizzard | Obstruction of gizzard |
| Prions | Salvin's prion | Soft plastic packaging | Gizzard | Obstruction of gizzard |
| Shearwaters | Short-tailed shearwater | Hard plastic | Gizzard | Obstruction of gizzard |
| Shearwaters | Short-tailed shearwater | Hard plastic | Isthmus and gizzard | Obstruction |
| Shearwaters | Short-tailed shearwater | Rope | Isthmus | Blockage |
| Shearwaters | Short-tailed shearwater | Balloon | Isthmus and proventriculus | Obstruction |
| Storm petrels | White-faced storm petrel | Hard plastic | Isthmus and gizzard | Obstruction |
| Fulmarine petrels | Southern fulmar | Soft plastic packaging | Gizzard | Obstruction of gizzard |
